# CSI-F: A Human Motion Recognition Method Based on Channel-State-Information Signal Feature Fusion

**DOI:** 10.3390/s24030862

**Published:** 2024-01-29

**Authors:** Juan Niu, Xiuqing He, Bei Fang, Guangxin Han, Xu Wang, Juhou He

**Affiliations:** 1 Key Laboratory of Modern Teaching Technology, Ministry of Education, Shaanxi Normal University, Xi’an 710062, China; 2023200348@snnu.edu.cn (J.N.); xiuqing@snnu.edu.cn (X.H.); beifang@snnu.edu.cn (B.F.); hgx@snnu.edu.cn (G.H.); mitm0829@snnu.edu.cn (X.W.); 2 School of Computer Science, Shaanxi Normal University, Xi’an 710062, China

**Keywords:** Wi-Fi channel state information (CSI), Doppler shift, direction-independent, cycle motion, recognition feature enhancement

## Abstract

The recognition of human activity is crucial as the Internet of Things (IoT) progresses toward future smart homes. Wi-Fi-based motion-recognition stands out due to its non-contact nature and widespread applicability. However, the channel state information (CSI) related to human movement in indoor environments changes with the direction of movement, which poses challenges for existing Wi-Fi movement-recognition methods. These challenges include limited directions of movement that can be detected, short detection distances, and inaccurate feature extraction, all of which significantly constrain the wide-scale application of Wi-Fi action-recognition. To address this issue, we propose a direction-independent CSI fusion and sharing model named CSI-F, one which combines Convolutional Neural Networks (CNN) and Gated Recurrent Units (GRU). Specifically, we have introduced a series of signal-processing techniques that utilize antenna diversity to eliminate random phase shifts, thereby removing noise influences unrelated to motion information. Later, by amplifying the Doppler frequency shift effect through cyclic actions and generating a spectrogram, we further enhance the impact of actions on CSI. To demonstrate the effectiveness of this method, we conducted experiments on datasets collected in natural environments. We confirmed that the superposition of periodic actions on CSI can improve the accuracy of the process. CSI-F can achieve higher recognition accuracy compared with other methods and a monitoring coverage of up to 6 m.

## 1. Introduction

As an important research topic affecting future intelligent life, the field of human activity recognition has matured, driven by the Internet of Things. People gradually transfer their interest in smart devices to infrastructure. Sensing, computing, and communication capabilities in an indoor environment allow people to “unplug” from their instruments while still receiving the same services available through portable or wearable devices. For example, with current lifestyle technology, counting a person’s steps requires that person to carry a device (smartphone or fitness band) with them, even at home. Similarly, tracking sleep behavior requires people to wear sleep-tracking devices while sleeping. The need to carry/wear the device creates significant discomfort for the user, and inaccurate measurements can occur when the user does not wear the device as recommended. With the advent of wireless utilization, such awareness and activity tracking can be achieved through Wi-Fi, alleviating the need for people to wear intelligent devices constantly. The technology can monitor a person’s activity schedule (such as sleeping or walking) using audio or video, but it poses privacy risks. However, recent research on activity tracking using radio frequency signals from wireless networks provides an attractive solution for device-free awareness. References [1,2,3] use Wi-Fi to achieve personnel positioning without equipment. Reference [2] calculates the position of the target personnel by Doppler estimation of the CSI signal. References [4,5,6] use Wi-Fi to realize device-independent motion perception. Wi-Fi provides a new method for indoor situational awareness due to its universality, low cost, and availability. Under IEEE 802.11n, the CSI of Wi-Fi devices further promotes the direction of device-independent activity identification.

Currently, Wi-Fi-based activity identification has many limitations. For example, most of the actions identified in the research work tend to involve facing the same direction, so it is not easy to ensure the robustness of recognition when users are facing different directions [7]. The forms of physical movement can often be recorded by cycles. For example, we perform behaviors in daily life such as walking, running, and doing fitness exercises. Cycle motion detection is an important research area within wireless sensing. With the periodicity of motion, motion features can be accurately captured and amplified to avoid the deviation caused by the detection direction, which provides a new solution for indoor human activity identification.

Using Wi-Fi to identify people facing different directions is a very challenging problem. An unrealistic and costly solution involves deploying multiple Wi-Fi devices indoors to detect movement from different directions. In addition, using existing Wi-Fi hardware to achieve fine-grained sensing with direction is complex and requires dedicated software, radio equipment, or antenna components [8]. This work aims to leverage the existing Wi-Fi infrastructure to achieve context-aware possibilities with low-cost deployment. To address these challenges, CSI-F relies on off-the-shelf Wi-Fi hardware devices and uses CSI to collect changes in Wi-Fi signals. Our study shows the specific steps involved in detecting a person’s motion directly from CSI after removing the influences of far multipath and noise. 

The action information in the signal domain is rich enough to describe the behavior of the human body. Previous studies have shown that unilateral movement can be detected in the human body. By analyzing a person’s actions, execution speed, and the overall changes in CSI caused by them, CSI-F can perceive the characteristics of each behavior from a series of actions. Another challenge is that, in real life, the scene of action recognition in a covered environment is not invariable, given such factors as interference of people and objects, change of indoor table and chair placement, etc., which would lead to different recognition effects of the same person’s action in the same environment based on CSI.

To solve the above problems, we propose a method called CSI-F, based on a Wi-Fi signal, to enhance the feature of human motion detection by taking advantage of the characteristics of cycle motion. CSI-F uses CSI to identify human movements and proves the orientation-independence of cycle movement recognition. Specifically, action CSI is collected by commercial Wi-Fi devices, and the discrete wavelet transformation (DWT) is used for noise reduction. Then, the principal components analysis (PCA) and the short-time Fourier transform (STFT) are used to extract the Doppler frequency shift of the motion data. Then, CSI-F builds a mixed action recognition model through deep learning networks such as Convolutional Neural Network (CNN) and Gated Recurrent Unit (GRU).

The contributions and novelty of CSI-F are as follows:

- Demonstrating the adaptability and sensitivity of CSI data to periodic movements and proposing a periodic feature slicing method. The method enhances the model’s recognition precision by superimposing periodic action CSI signals, which could offer more application scenarios for context-aware computing, such as device-free indoor pedometers, fitness activity counters, and more.
- The direction-independent nature of the CSI-F method has been validated. The dependency of human motion recognition methods on direction has been resolved using Doppler characteristics and antenna selection techniques. Experiments demonstrate that the direction of movement does not significantly affect the experimental results.
- A multi-task feature-sharing and information fusion model is proposed. Combining CNN, GRU, and multi-task learning facilitates information sharing and specific information fusion among tasks. The data-driven components in this hybrid model structure can identify complex non-linear relationships between the temporal and spatial layers of the input. Moreover, the model’s generalizability and predictive accuracy are effectively enhanced through coordination and assistance among tasks.
- In most cases, the indoor motion-sensing of individuals can be recognized within two to three cycles, enhancing the accuracy and usability of Wi-Fi signal perception. Additionally, the periodic nature of the movements allows for the expansion of the sensing distance to up to 6 m.

In Section 2, we describe the related work. In Section 3, we introduce the scheme’s basic knowledge and preliminary work. In Section 4, we present the proposed recognition method. In Section 5, we introduce experiments and discuss the results. In Section 6, we summarize our work.

## 2. Related Work

### 2.1. Action Recognition Method

At present, human motion recognition is based on a variety of technical schemes. We will elaborate on relevant work from the two categories of contact action recognition and non-contact action recognition and describe in detail the domestic and foreign research progress in motion recognition based on CSI. At the same time, to show the difference between the relevant work and the method in this paper, as well as the advantages of the method in this paper, Table 1 and Table 2 are set up for specific descriptions.

#### 2.1.1. Contact Action Recognition Method

Sensor-based technology has been widely used in the field of human motion recognition. Among these technologies, the primary hardware devices are smartphones and wearable devices. In reference [9], the authors have collected human sports activity data from three-axis accelerometers and explored high-precision recognition methods based on deep learning. Reference [10] applies sensors embedded in smartphones to collect human activity data, achieving high-precision recognition based on neural-network models. Reference [11] uses multiple wearable sensors (such as accelerometers, magnetometers, etc.) to read human movement data. It uses the Recurrent Neural Network (RNN) network to identify running, sitting, standing up, cycling, and other movements, proving that our method has an effective recognition capacity. Such techniques require users to collect data in a wearable way, which not only causes inconvenience to users in all its aspects but also brings privacy and security problems.

#### 2.1.2. Non-Contact Action Recognition Method

Non-contact human motion recognition is divided into computer vision and wireless perception technology. Reference [12] provides a comprehensive summary and analysis of video-based human motion recognition methods and discusses their current development status. The authors achieved human activity recognition by performing convolution on the video streams in reference [13]. The Long Short-Term Memory (LSTM) network is used for video action recognition, based on the attention mechanism. The computer vision method cannot work in weak light or in a dark environment. The perception method of radio frequency signals overcomes the limitation of computer vision. Considering the equipment collecting wireless signals, the research focuses on motion recognition, which is mainly distributed in radio frequency identification (RFID), ultra-wideband (UWB) radar, and Wi-Fi [14]. Reference [15] designed an RFID-based human head gesture recognition system which collects received signal strength to capture changes in head gestures. In reference [16], UWB radar was used to extract Doppler images, the PCA algorithm was used to extract features, and then GRUs were used to classify and recognize actions. However, all of the above require dedicated equipment to collect data and are complex to deploy. 

In reference [17], Yu Gu et al. proposed an RSS-based indoor activity recognition framework which uses a fusion algorithm to classify and identify daily indoor activities. However, the measurement of RSS is volatile due to the multipath effect and channel fading, and the large amount of environmental noise carried by RSS is also arduous to deal with. Compared with RSS, CSI better reflects the information of the physical layer in the signal transmission process, can better reflect the changes in wireless signals, and has a finer identification granularity [18,19]. Recently, Zhang et al. solved the problem of individual inconsistencies in human activity recognition through the eight-channel-state-information (CSI) conversion method. Previous studies used relatively small amounts of activity data for recognition, so various activity data were integrated to solve the problem of different CSI signals for other individuals [20]. Gu et al. designed a system incorporating activity recognition and monitoring to achieve fine-grained activity recognition [21]. However, none of these methods can be directly used in situational perception due to their shared inability to adapt to different scenes.

**Table 1:** The differences between CSI-F and related technologies.

Technology	Related Literature	Research Object	Equipment	Advantage	Disadvantage
Sensor-based technology	Reference [9]	Human motion data	Triaxial accelerometers	High-precision detection, which can fully and accurately reflect the motion properties of objects.	Troublesome to wear, and poor privacy.
	Reference [10]	Human motion data	Smart phone built-in sensor		
	Reference [11]	Human motion data	Wearable accelerometer		
Computer vision	Reference [12]	Human motion data	Camera	Reliable data, real-time monitoring, fast information processing, non-contact.	Poor privacy, greatly affected by light and other factors.
	Reference [13]	Human motion data	Camera		
RFID	Reference [15]	Head gesture data	RFID tags	Reliable data, real-time monitoring, fast information processing, non-contact.	Short operating distance (generally in the scale of tens of meters) and poor privacy.
UWB	Reference [16]	Human motion data	UWB	High positioning accuracy, short working time and low power consumption.	There should be no obstacle to block the radio transmission, and there should be a perfect positioning network.
Wi-FiRSS	Reference [17]	Human motion data	AROCOV 6260 ap	No contact, no need for equipment, privacy.	Vulnerable to the impacts of dynamic environments, and difficulties in dealing with environmental noise.
Wi-FiCSI	CSI-F	Human motion data	Intel 5300 network card	It can overcome the shortcomings of all the above technologies, and is cost-effective, with strong privacy and wide coverage.	The recognition of micro-motions in harsh wireless environments is limited, and the optimization of the model is not perfect.

**Table 2:** The differences between CSI-F and related work of action recognition based on Wi-Fi CSI.

Related Work	Object	Equipment	Method	Advantage	Shortcoming
Reference [19]	Human activity data	Intel 5300 network card	Bidirectional Long Short Term Memory Network BiLSTM	Recognition of human activities by models in various experimental scenarios, with a recognition accuracy of 90%.	The generalization ability of the model and the recognition accuracy of the model need to be improved
Reference [20]	Human activity data	Intel 5300 network card	LSTM	By integrating multiple CSI data from various activities, the impact of inconsistent activities and certain topic-specific issues were eliminated.	The mobility and universality of motion-recognition methods are not considered.
Reference [21]	Human motion data	Intel 5300 network card	A dual-channel neural network	It can accurately classify various human activities, with an average accuracy rate of 93.3%.	Mobility in multiple environments without considering action recognition methods.
CSI-F	Human daily motion data	Intel 5300 network card	GRU + CNN	Improves the generalization ability and prediction accuracy of the model through information enhancement and multi-task sharing.	No discussion as to finer-grained actions.

### 2.2. Human Activity Perception Principle

**Figure 1:**
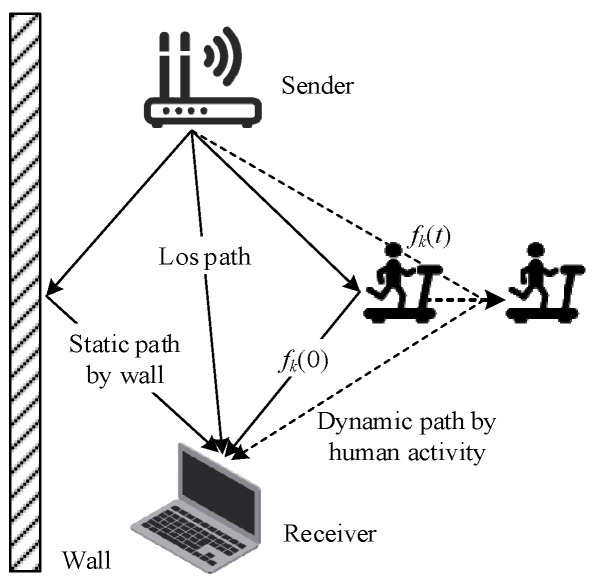
Multipath effect caused by human movement.

When Wi-Fi signals are transmitted indoors, they are affected by static obstacles such as tables, chairs, and walls, resulting in multi-level propagation paths called static paths. Dynamic paths will be generated when dynamic objects or people move in a Wi-Fi environment, as shown in Figure 1. Compared with the line of sight (LOS), the arrival time of signals in the reflected path is different, so the receiver will receive signals from multiple paths successively, which is called the multipath effect [22].

The logarithmic path-loss model estimates the relationship between average path-loss and propagation distance. The middle path-loss $\bar{Lp}(d)$ can be expressed as Equation (1):

(1) $$\bar{Lp}(d)=\bar{Lp}(d_{0})+10\eta \mathrm{lg}(\frac{d}{d_{0}}),d\geq d_{0}$$

where $d_{0}$ is the reference distance, the typical indoor value is 1 m, $\bar{Lp}(d_{0})$ is the reference path-loss, and η is the path-loss factor, which describes the change rate of the path-loss distance. In free space, η is usually 2. When the shadow effect is considered, the expression of path-loss can be expressed by Equation (2):

(2) $$Lp(d)=\bar{Lp}(d_{0})+10\eta \mathrm{lg}\left(\frac{d}{d_{0}}\right)+\varepsilon ,d\geq d_{0}$$

ε is the variable of the shadow effect, and its value follows a Gaussian distribution. If wall *W*, ceiling *C*, and solid furniture *S* are affected by a typical indoor environment, Equation (2) can be further written as

(3) $$Lp(d)=\bar{Lp}(d_{0})+10\eta \mathrm{lg}\left(\frac{d}{d_{0}}\right)+\varepsilon +\sum_{i=1}^{N}W_{i}+\sum_{i=1}^{N}C_{i}+\sum_{i=1}^{N}S_{i},d\geq d_{0}$$

where $\bar{Lp}\left(d_{0}\right)+\sum_{i=1}^{N}W_{i}+\sum_{i=1}^{N}C_{i}+\sum_{i=1}^{N}S_{i}$ can be regarded as a constant in the interior, so Equation (3) can be further rewritten as

(4) $$Lp\left(d\right)=10\eta \mathrm{lg}\left(\frac{d}{d_{0}}\right)+\varepsilon^{\prime },d\geq d_{0}$$

Thus, the received signal power $P_{r}\left(d\right)$ of the receiver can be obtained from Equation (5), below.

(5) $$P_{r}\left(d\right)=P_{t}-Lp\left(d\right)$$

By substitution of $Lp\left(d\right)$ in Equation (5), one can obtain:

(6) $$P_{r}\left(d\right)=10\eta \mathrm{lg}\left(\frac{d}{d_{0}}\right)+\varepsilon^{\prime \prime }$$

Receiver signal power $P_{t}$ is also a constant, $\varepsilon^{\prime \prime }$ follows a Gaussian distribution with an average value of μ, and μ is related to transmitting power and path loss. Thus, it can be seen that the transmitter power is affected by static factors. When the human body moves, Equation (6) becomes Equation (7):

(7) $$P_{r}(d)=10\eta \mathrm{lg}(\frac{d}{d_{0}})+\varepsilon^{\prime \prime }+\Delta$$

Δ is an approximate dynamic change in path length caused by the human body. As a result of this change, the human body will interfere with the signal when it moves, changing the power of the received signal accordingly. 

We use CSI extracted from Wi-Fi signals to recognize human movements. Under the IEEE 802.11n protocol, CSI can be removed from Wi-Fi signals using orthogonal frequency division multiplexing (OFDM) technology [23]. In the propagation of wireless signals, the propagation channel is usually represented by the channel frequency response (CFR):

(8) $$H\left(f\right)=\sum_{k=1}^{R}\alpha_{k}\cdot e^{-j\cdot 2\pi \cdot f\tau_{i}}$$

where R is the number of paths, $\alpha_{k}$ is the amplitude information of link *k*, $e^{-j\cdot 2\pi \cdot f\tau_{i}}$ is the phase difference, $\tau_{i}$ is the propagation delay, and f is the carrier frequency. In order to obtain the power delay distribution, inverse fast Fourier transform (IFFT) can be used to convert the CFR into the channel impulse response (CIR) [24]:

(9) $$h\left(\tau \right)=\sum_{i=1}^{N}a_{i}e^{-j\theta_{i}}\delta \left(\tau -\tau_{i}\right)$$

where $a_{i}$ is the amplitude attenuation of path *i*, $\theta_{i}$ is the phase offset of path *i*, and $\tau_{i}$ is the delay of path *i*. *N* is the total number of multipaths, and δ is the impulse function. On a certain subcarrier, CSI can be expressed by the following equation:

(10) $$H_{k}=|H_{k}|\cdot e^{j\sin\theta_{k}}$$

where $H_{k}$ is the CSI function of the *k*th subcarrier, $\left|H_{k}\right|$ is the amplitude of the *k*th subcarrier, and $e^{j\sin\theta_{k}}$ is the phase information. When people perform corresponding actions, the amplitude and phase reflected by CSI will change accordingly. Exploring the laws governing its change can help to achieve effective human action recognition.

## 3. Data Preprocessing

### 3.1. Phase Preprocessing

**Figure 2:**
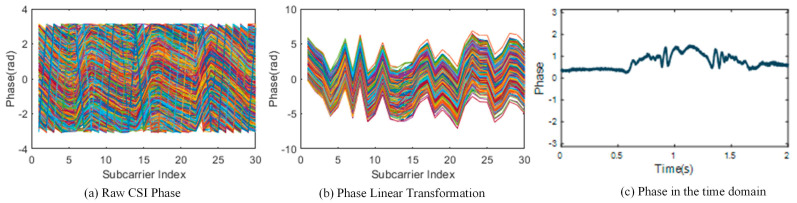
CSI phase-processing. Each line of different colors represents the amplitude variation curve of the subcarrier on the nth data packet.

In wireless communication, the center frequency, sampling frequency offset, environmental noise, and hardware noise are the leading causes of CSI phase offset. The phase offset value of the same subcarrier received by the same antenna is stable [23]. To enhance the sensitivity to CSI changes caused by human actions, it is necessary to eliminate the phase deviation of CSI to better balance the static response and dynamic response. CSI with a high amplitude usually has a sizeable static reaction due to strong LOS signals in the indoor environment, such as reflective objects like the flat surfaces on desks and chairs. Such variance helps the feedback as to the impact of action changes on CSI, so that it can better reflect dynamic responses. Therefore, two antennas with the maximum amplitude and minimum variance of CSI have been selected in CSI-F to calculate the conjugate matrix of these two antennas in order to amplify the dynamic component and reduce the static influence from different directions, and better extract the Doppler features. The phase after linear transformation is shown in Figure 2a,b, and the phase of the reaction in the time domain is shown in Figure 2c.

### 3.2. Amplitude Information

**Figure 3:**
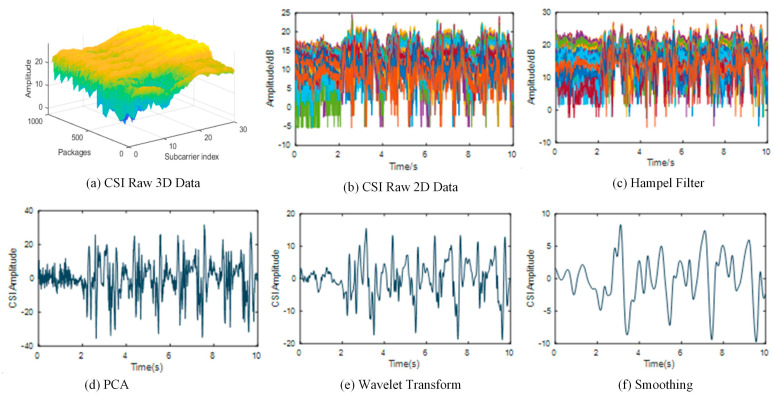
CSI wavelet transform diagram. In (**a**), different colors represent different channels, and there are a total of three channels. Each line of different colors represents the amplitude variation curve of the subcarrier on the nth data packet in (**b**,**c**).

In the original data, low-frequency interference and burst noise are unrelated to the action. Therefore, to retain CSI signals from human activities accurately, Hampel filtering was used in our study to reduce the interference of hardware or other surrounding factors with the action information. After PCA transformation and DWT, high-frequency noise information higher than human movement was filtered out for the first time, and a Butterworth filter was used to reduce noise. CSI data before and after processing are shown in Figure 3.

Figure 3a is the originally extracted three-dimensional CSI data, and Figure 3b is the two-dimensional CSI information in the time domain. Figure 3c is the waveform after Hample filtering. The subcarriers retain the period and peak transformations caused by human activities. It can be seen that the filtered CSI is good enough to maintain the peak value and mutation part reflecting the human motion state in the original signal and can effectively remove the high-frequency burr caused by the multi-path environment and narrow-band influence. To further extract stable Doppler frequency shift features using STFT changes, we have used the principal component analysis method to select subcarriers, and the selection results are shown in Figure 3d. To remove noise interference, a wavelet change scheme is introduced to process subcarrier data, as shown in Figure 3e. The final results obtained after smoothing are shown in Figure 3f.

### 3.3. Cyclicity Segmentation

**Figure 4:**
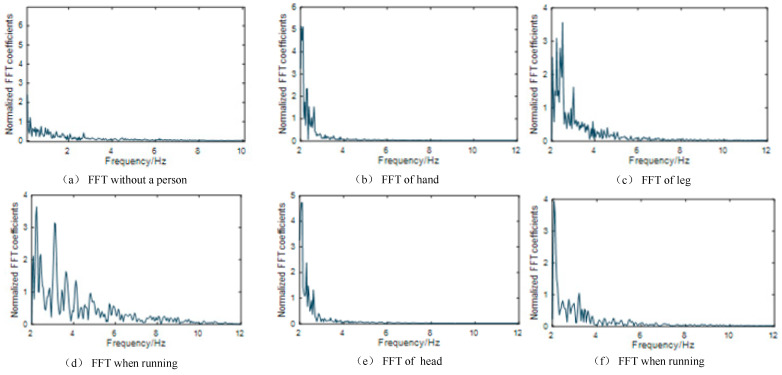
FFT transformation curve.

Because of the cycle change of fitness exercises, walking, and running, the movement recognition should accurately distinguish the beginning, transformation, and end of the movement. Reference [25] has proved that different behavioral activities of personnel will cause differences in energy intensity and frequency. To capture the motion, we set up an energy indicator to realize the motion segmentation scheme. When no movement occurs in the Wi-Fi area, we can see the fast Fourier transform (FFT) curve in Figure 4a, and the fast Fourier transform curve of the experimental subject’s leg movement behavior is apparent in Figure 4b. Compared with the change in energy caused by leg movement, the energy that occurs without movement is lower. Therefore, the energy indicator detects the FFT transformation value of CSI according to the action CSI sequence after denoising.

(11) $$E_{w}=\sum_{i=1}^{n/2}mag^{2}$$

where $E_{w}$ is the energy, n is the length of the time window, and mag is the normalized FFT coefficient calculated within the time window, per second.

**Figure 5:**
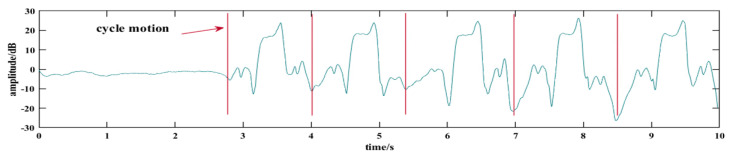
Cycle activity segmentation curve.

The cycle motion after energy segmentation can be easily captured by this method, as shown in Figure 5. CSI-F monitors the difference in short-term motion energy in two continuous windows. CSI-F considers that action occurs when the difference is more significant than the positive threshold. When the action is completed, the motion energy in the window drops sharply; that is, the difference is less than the negative threshold. Thus, the feature map containing only one action can be correctly segmented during action conversion.

Thus, CSI-F disassembles each cycle action through the sliding window, and the disassembled action is superimposed according to the time interval. After multiple signals are superimposed, the average value is taken to obtain the new continuous action signal, as shown in Equation (12).

(12) $$\overset{⌢}{H}\left(f,t\right)=\frac{1}{N}\sum_{i=1}^{N}H\left(f,t_{i}\right)$$

## 4. Methods of CSI-F

### 4.1. Overview

**Figure 6:**
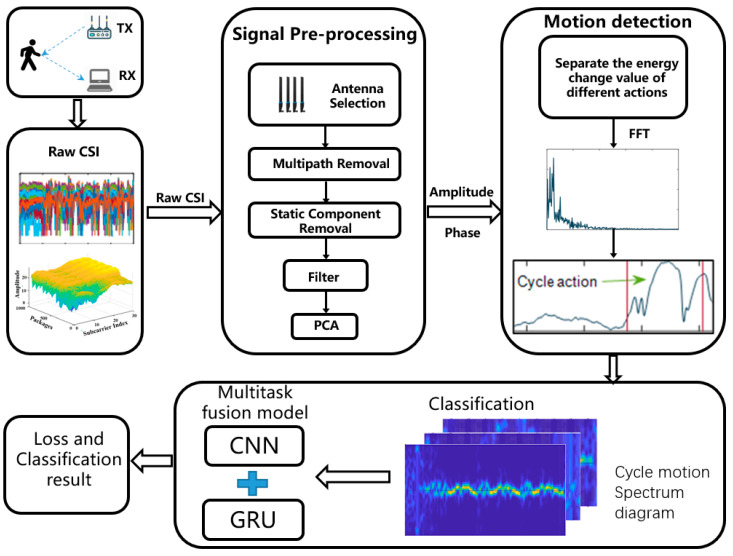
Overall flow chart of the CSI-F model.

CSI-F uses invisible Wi-Fi signals to identify human movement; Figure 6 shows an overview of CSI-F. The workflow of CSI-F is divided into three steps: data acquisition, data processing, and feature extraction. Two experimental devices, each equipped with an Intel 5300 NIC, are deployed to collect human motion data in the scenario. Here, we use the Intel 5300 Monitor mode to complete the acquisition of CSI motion data.

### 4.2. Data Processing and Feature Extraction

We conducted a principal component analysis on the CSI data to further denoise and reduce the dimensionality of the CSI data to extract actionable information for time–frequency analysis. The first central component contains the main power change caused by motion, which is selected as the input of the short-time Fourier transform (STFT) to extract the Doppler frequency shift feature. When the human torso moves towards the transmitter and receiver, the peak and trough of the reflected electromagnetic wave signal reach the receiver faster. In contrast, when it is far away, the peak and trough run to the receiver at a slower speed. In general, the Doppler shift can be expressed by Equation (13):

(13) $$D_{f\left(i\right)}=-\frac{1}{\lambda }\frac{d}{d_{t}}d\left(t\right)$$

λ is the wavelength of the signal, and d(t) is the length of the reflected path. According to Equation (8), the Doppler frequency shift of CSI can be obtained by time–frequency analysis of the paired spectrum:

(14) $$H\left(\tau \right)\approx h\left(\tau \right)+\sum_{n\in D}a_{i}B\left(f_{D_{n}}\left(t\right)\right)$$

$B(f_{D_{k}}(t))$ is the window function for intercepting the CSI action signal segment. Unknown phase shifts in the original CSI have been caused by the lack of synchronization between Wi-Fi network cards:

(15) $$\tilde{h}\left(\tau \right)=h\left(\tau \right)e^{-j2\pi \left(\Delta_{t}f+\Delta_{f}t\right)}$$

$2\pi \left(\Delta_{t}f+\Delta_{f}t\right)$ is the carrier frequency and time offset caused by the phase shift. Therefore, extracting the Doppler component directly from the actual CSI is not feasible. We use a different antenna on the Wi-Fi network card to solve this problem and eliminate the unknown phase-shift while preserving the complete Doppler shift. Antennas from the same network card all have the same phase-offset, and the phase offset is eliminated by calculating the conjugate multiplication of the CSI of a pair of antennas [26]. This study obtains a stable frequency-domain feature, namely Doppler frequency shift, from the CSI data collected by short-time Fourier transform. Because of the characteristics of Doppler frequency shift, it can better reflect the changes of human movement in the frequency domain, thus weakening the influence of static factors, including direction, on the CSI characteristic information related to action.

CSI depicts the influence of human movement on channel state in the frequency domain, and specific actions can be identified only by establishing an effective mapping between CSI and human movement. The pre-processing stage of data is critical and determines the generalization ability of a recognition model. However, due to the severe multipath effect and other environmental components in the indoor environment, the obtained data contains a significant amount of noise, which affects the effectiveness of subsequent feature extraction and the accuracy of motion-recognition. We usually choose effective data processing methods according to the categories of motion features and the ranges of energy variation of the human body in the frequency domain.

**Figure 7:**
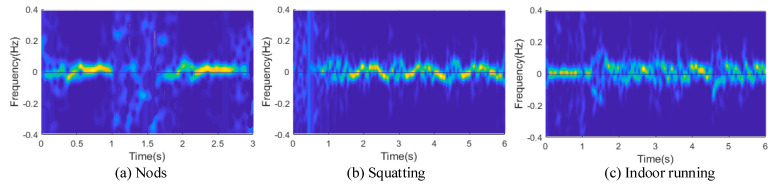
Doppler spectrogram of CSI.

In addition, we built a frequency domain energy indicator to judge as to the starts and stops of human motion and enhance the system’s usability. We took the Doppler frequency shift and FFT values of human motion in the frequency domain as the standard motion-recognition features. This feature effectively reduces the influence of motion direction information, judges the start of motion, and has good environmental migration and recognition ability. In an indoor situation, human movement is changeable, so there are generally two or more energy transformations in the spectrum map generated by the time–order relationship. The image recognition method using local search can distinguish different actions, but it will increase the burden of labeling and system overhead. To reduce the time complexity of the model, we need to use the global search strategy, so a graph must be a kind of action, which requires us to divide the different actions first when generating the spectrum graph. Therefore, the energy indicator helps us to solve this problem, and the schematic diagram of the Doppler frequency shift after segmentation is shown in Figure 7.

Figure 7 shows CSI’s Doppler frequency shift spectrum, as caused by head, hand, and leg movements, provided by commercial Wi-Fi network cards. Figure 7a describes an instance of nodding behavior across 3 s. From 0–1 s, the bowing movement occurs, and 2–3 s is the raising movement. Figure 7b shows the spectrum transformation caused by squatting. Figure 7c shows the Doppler frequency shift caused by people running indoors. Although the energy of each Doppler shift fluctuates, the figure can reflect the Doppler effect caused by the change of direction and action and has an obvious periodicity, which provides a reliable data source for model classification and an effective passive period-counting method. At this point, cycle actions are thoroughly divided.

### 4.3. Human Activity Recognition Using CSI

In this study, we use an inductive transfer mechanism to improve the generalization ability of the action recognition model and build a shared layer to interpose the domain-specific information between the target action and the actions CSI features in its related scenes to improve the generalization. Through the deep CSI extracted from multi-layer features to describe the relationships between tasks, it has a more robust feature-learning ability.

**Figure 8:**
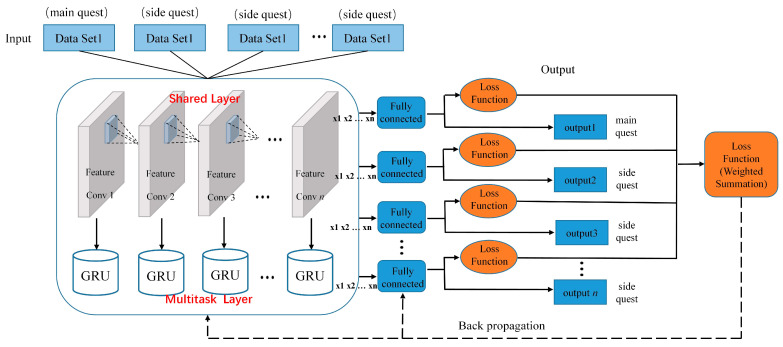
Action recognition model.

A convolutional neural network can obtain more spatial features. GRU, as a time-deep neural network, can solve the long-term dependence problem of time series [27]. Therefore, the Convolutional Neural Network and Gated Recurrent Unit hybrid model can be applied to motion recognition to extract better temporal and spatial features. Thus, CSI-F will construct a multi-task deep-learning model for action recognition. The model uses multiple related tasks to share the network structure and uses CSI data with strong correlation as auxiliary data to optimize and improve the learning performance of target action-recognition tasks. The model is divided into three modules: the sharing, specific task, and joint optimization layers. The framework of the action-recognition model is shown in Figure 8. Table 3 shows the super-parameters of the three models.

#### 4.3.1. Shared Layer

The learning and sharing of typical action CSI data features occurs through the sharing layer. The CSI data of multiple related motion-recognition tasks are input to a CNN for training to obtain a general parameter model, which can not only share parameters among multiple prediction tasks but also reduce the risk of overfitting for each recognition task. CSI data of the same action in different scenes have strong temporal and spatial correlations. In the sharing layer, the branch task needs to provide spatiotemporal information for the main task so that the main task can better extract the spatiotemporal features of the CSI data. A CNN can cut down the complexity of the network model, has a strong mentality, and has a formidable data-processing function. Using the CNN as the sharing layer of the recognition model can effectively catch the strong-feature correlation between motion-based CSI data in different scenes. At the same time, the parameter sharing of the shared layer of each task and the weight sharing of each CNN convolution layer complement each other. The main task is to learn the CSI related to the actions in the branch mission.

For fear of the loss of feature information caused by the CNN pooling layer and the loss of position information that the entire connection layer may cause, the convolution layer of the CNN is directly used as the sharing layer of the recognition model. CSI action data in multiple scenarios are used as numerous tasks of the model, and the action CSI data of each task is input to the CNN for calculation. The feature map $X^{l-1}=\left\{x_{1}^{l-1},x_{2}^{l-1},\cdots ,x_{i}^{l-1}\right\}$ output by the previous layer (*l* = 1 represents the input of the model) is convolved by filter $\left\{k_{1j}^{l},k_{2j}^{l},\cdots ,k_{ij}^{l}\right\}$ at the *l* convolution layer to obtain the net activation quantity $v_{j}^{l}$ of the *j*th channel:

(16) $$v_{j}^{l}=\sum x_{i}^{l-1}*k_{ij}^{l}+b_{j}^{l}$$

$B_{j}^{l}$ is the deviation. Then, $v_{j}^{l}$ of the *j*th channel is used to calculate the output characteristic map $x_{j}^{l}$ of the *j*th channel in the *l* convolution layer through the ReLU activation function:

(17) xjl=ReLUvjl=max0.vjl

Finally, the multi-channel output feature map $X^{l}=\left\{x_{1}^{l},x_{2}^{l},\cdots ,x_{j}^{l}\right\}$ serves as the input of the next convolution layer.

#### 4.3.2. Multitask Layer

Learning of the long-term dependence characteristics of the action CSI in each scene within the same experimental environment occurs through the multitask layer. For each recognition task, the time-series characteristics of action CSI are learned through multiple GRU training instances to ensure the difference of each action recognition. Each identification task obtains the identification result through the entire connection layer.

CSI data in different scenarios have differences in time series. The specific task layer constructed by the GRU is used to extract the time series features of CSI data. Each recognition task is trained by multi-chamber GRU and its feature vector is obtained. Finally, the recognition result is converted by the GRU feature vector, decoded by a fully connected layer. The feature map *X^l^* output by each task in the shared layer is reconstructed into a two-dimensional vector *x^t^*. Then *x^t^* and the previous memory content *h_t_*_−1_ are spliced and input to the GRU. The update gate *z_t_* and reset gate *r_t_* of GRU are calculated through the sigmoid function σ:

(18) $$z_{t}=\sigma \left(W^{z}x_{t}+U^{z}h_{t-1}\right)$$

(19) $$r_{t}=\sigma \left(W^{T}x_{t}+U^{T}h_{t-1}\right)$$

where *W* is the input to the implicit weight matrix and *U* is the recursive weight matrix from state to state. The value of *z_t_* is used to determine which CSI signal data to retain, and reset gate *r_t_* is used to determine which signals to discard.

In order to remember the current state, the relevant CSI is stored as the product of *r_t_* and *h_t−_*_1_, and then splitted with *x_t_*, and the new candidate memory capacity ${\overset{⌢}{h}}_{t}$ is calculated by activation function tan*h*:

(20) $${\overset{⌢}{h}}_{t}=\tanh\left(Wx_{t}+U\left(r_{t}⊗h_{t-1}\right)\right)$$

where ⊗ is element-level multiplication. Afterwards, the current memory substance *h_t_* can be expressed as a combination of selective forgetting of circumstantial information and selective retention in memory of important information:

(21) $$h_{t}=\left(1-z_{t}\right)⊗h_{t-1}+z_{t}⊗{\overset{⌢}{h}}_{t}$$

The values 1 − *z_t_* and *z_t_* can complement each other and remain constant. In the task-specific layer constructed by GRU, the long-term dependency features in the time series of each recognition task’s historical input CSI data are captured by the update gate *z_t_*, and the short-period dependency is captured by the reset gate *r_t_*. Different GRUs were trained for the main and the secondary tasks, which not only extracted the long-period dependence characteristics as to time series in the tasks but also ensured the differences between tasks in different scenarios.

Multiple loss functions are obtained through the output and actual values of numerous tasks. Then, the weighted sum of all losses is taken as the total loss of the model, and the backpropagation algorithm is used to optimize the model. Finally, the recognition results of multiple tasks are obtained through iterative training.

This optimization mechanism combines weighted loss function and backpropagation, which plays a role in feature sharing and information fusion between tasks. The interaction between tasks can keep the feature-learning process from falling to a local minimum. We use the deep CSI feature information learned from the deep neural network to describe the relationships of action signals in different scenes and achieve the purpose of information sharing by processing the parameters of specific network structures. There result *N* prediction missions, consisting of main and side missions. The training set *D_n_* of task *n* can be expressed as

(22) $$D_{n}={\left\{\left(x_{t}^{n},{\overset{⌢}{y}}_{t}^{n}\right)\right\}}_{t=1}^{S_{n}}$$

where $x_{t}^{n}$ is the *t*th sample of task *n*, and *Sn* is the number of samples of task *n*. ${\overset{⌢}{y}}_{t}^{n}$ is the corresponding label output.

The proposed model improves action-recognition performance through action-recognition tasks in different scenarios. The standard features of CSI data of the same action shared between different scenarios are learned through sharing, and then heterogeneous features are known through a task-specific layer. Finally, the multi-weighted losses of action recognition tasks in different scenarios are summed to obtain the overall loss of the model, and the joint optimization of the model is realized by backpropagation. The loss of task *n* is defined as the least mean squares form:

(23) $$Loss_{n}=\frac{1}{S_{n}}\sum_{t=1}^{S_{n}}{\left(y_{t}^{n}-{\overset{⌢}{y}}_{t}^{n}\right)}^{2}$$

where $y_{t}^{n}$ is the identification result of the model, due to the different levels of importance of the main and secondary tasks in the recognition model, as well as the characteristics of each task. The overall loss of the recognition model can be defined as the weighted sum *Loss_n_* of each loss:

(24) $$Loss=\sum_{n=1}^{N}\beta_{n}Loss_{n}$$

where $\beta_{n}$ is the weight of task *n*. The parameter-sharing mechanism and multi-loss joint optimization method in the model proposed in this paper can take into account the action-recognition tasks from different angles in the same experimental environment and avoid the risk of over-fitting to a certain extent. After iterative joint optimization, the final trained model can be used for a CSI-based action recognition model and can realize cross-scene recognition and an environment-transfer recognition mechanism in the same experimental environment.

#### 4.3.3. The Training Process of the Model

CSI action data collected in this study are divided into training and test sets. The training set adopts the data contained in different scenarios (EA, EB, EC, and ED, as in Section 5.1) in an open hall environment, and the test set adopts CSI data collected in different experimental scenarios in a laboratory or conference room environment. The model is trained through the training set, and then the test set evaluates the recognition model. The specific steps of CSI-F are as follows:

Step 1: Entry of a value. The action recognition for each scene is defined as a task. The recognition of the action in the target scene is called the main task, and the recognition in other scenes in this environment is called the secondary task. For example, when EC is the main task, the EA, EB, and ED actions are identified as side tasks.

Step 2: Learning to share CSI features. The CSI data of multiple tasks are input to the shared layer at the same time. We use the convolution layer of a multi-layer CNN to extract features. Numerous tasks can share parameters in a shared layer. Finally, we will excavate some standard action features that represent multiple scenes.

Step 3: Learning about heterogeneous features. We input the action features learned from the sharing layer to the task layer for further processing. Each task uses different GRUs to understand the long-term dependence characteristics of each time series. After that, we can sufficiently identify the task-specific information of each task in the time series.

Step 4: Calculation of the prediction error. The loss associated with each task is calculated according to the output of each task and the actual category (marked in advance). Then, we calculate the global loss of the prediction model through the weighted sum of all task losses. Finally, we can use the global loss to correct the model error.

Step 5: Model optimization. We use the backpropagation algorithm to iterate the training set many times to optimize the parameters of the prediction model.

Step 6: Output. After several iterations of training, we can obtain the final parameters of the model.

The algorithm corresponding to the above training process is outlined in Algorithm 1.
**Algorithm 1:** Motion recognition methodInput: training data set *D_n_* (1 < n < *N*); The upper limit *I* of the learning time; Learning rate a initialize all adjustable parameters $\theta_{0}$
Calculate *D_n_* by Equation (10)for i to *I* do  for n = 1 to *N* do    $X^{l}=CNN_{shared}\left(D_{n}\right)$    $y_{t}^{n}{=\mathrm{GRU}}_{n}(X^{l})$
    Calculate *L_n_* by Equation (10)  end for  Calculate *L* by Equation (12)  $\theta_{i}\leftarrow \theta_{i-1}-a\nabla_{\theta }L\left(\theta \right)$
  If *L* stops decreasing after ten times then     break   end ifend for

## 5. Experimental Design and Analysis

### 5.1. Experimental Setup

The experiment used an Intel^®^ Core™ I7-7800X CPU, 3.50 GHz, 64.00 GB memory (Intel, Santa Clara, CA, USA), and an Nvidia GeForce GTX 1080 Ti GPU (Nvidia, Santa Clara, CA, USA). We divided the hardware equipment used in the experiment into the receiving and transmitting ends. The receiving and transmitting ends are two computers with Intel 5300 network adapters. The wireless network adapter is connected to three external omnidirectional antennas. The CSI tool is used to extract CSI information from the network adapter. The distance between the transmitter and receiver is 6 m. Three transmitting antennas and one receiving antenna were set up in the experiment, and three transmission links were used. The channel’s center frequency was set to 5.7 GHz, and the sampling rate adjusted to 512 Hz. The TensorFlow framework is adopted in this paper. In the fine-tuning process, training and validation sets are randomly selected from the data set. The initial learning rate was 0.001 for every two epochs, multiplied by 0.94.

**Figure 9:**
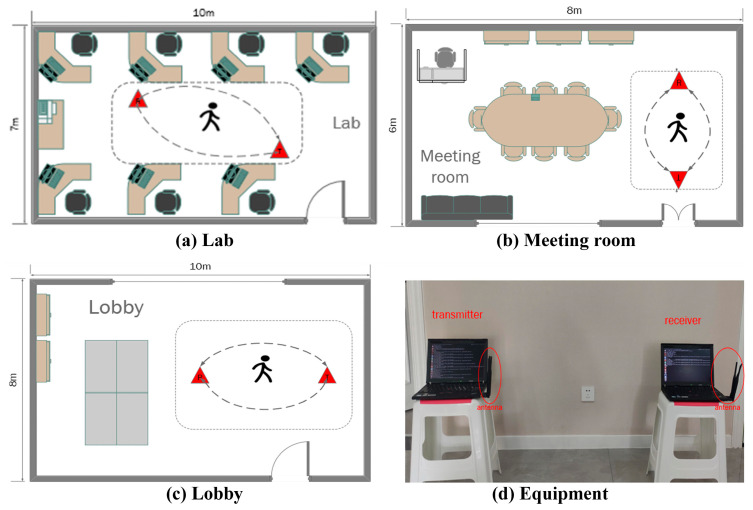
Plan of the experimental scene and the experimental device.

To verify the environmental mobility of the model, three experimental environments were set up to collect empirical data: a laboratory, a conference room, and an empty indoor hall. The laboratory area is surrounded by office desks, chairs, and other equipment, which is a complex environment. The conference room is equipped with round tables and chairs, a semi-empty setup compared to the classroom. The hall is an empty area. The experimental movements were divided into four categories (head, hand, leg, and torso), comprising ten movements. The head movements included two movements, nodding and shaking the head; the hand movements included left and right waves, as well as up and down motions; the leg movements included walking and running; the torso movements consisted of a series of indoor fitness exercises comprising push-ups, squatting, sit-ups, and jumping, divided into four movements. The above action data were collected in the three different environments in the experiment; the schematic diagram of the scene is shown in Figure 9.

### 5.2. Experimental Factor Analysis

#### Overall Accuracy

**Figure 10:**
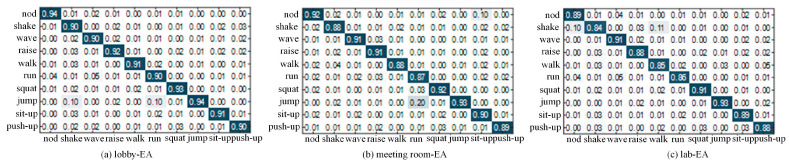
Identification accuracy in three scenarios. In the confusion matrix, darker colors are used to highlight the probability of being correctly classified.

In this section, we will evaluate the Wi-Fi, in general, by using a typical Wi-Fi device. From among the data obtained, the data collected in the EA, EB, EC, and ED scenarios in the empty hall were used for training. The data were therefore collected in the same environment. Settings in the laboratory and conference room were used for testing. Figure 10, a confusion matrix, shows the overall perception-recognition rate for ten daily behaviors in EA perception scenarios in the hall, conference room, and laboratory environments.

It can be seen that CSI-F has excellent performance in different experimental environments. The experimental results show that CSI-F performs best in the open hall, and its overall average accuracy is 92%, 91%, 90.5%, and 92%, respectively, when the movements occur in the head, hands, legs, and body. CSI-F performs worst in the laboratory scenario, because there are many obstacles and static components in a laboratory, leading to severe multipath effects and reduced accuracy of motion-recognition. However, when people are in an empty hall, there are almost no obstacles to block. Hence, the motion signal is relatively complete, and the recognition accuracy of the motion is improved accordingly. In an office scenario, where static and dynamic components are balanced, the overall average recognition rate of actions is 91.75%, which fully demonstrates the robustness of CSI-F for common indoor scenarios.

**Figure 11:**
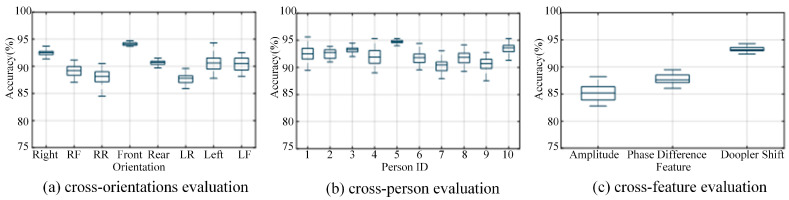
Accuracy distributions for different variables.

In the cross-domain evaluation, we will evaluate CSI-F in the same environment and in different fields, mainly including the identification accuracy of different directions, personnel, and feature methods. To make a reasonable evaluation of each sub-field, this paper adopted the control technique in the experimental process: only changing a single variable to keep other factors consistent. Figure 11 shows the recognition accuracy in three scenarios.

1. Orientation diversity

To verify whether CSI-F solved for the influence of the difference in each action’s direction on the action-recognition method based on CSI, this paper set up eight identical actions in different directions, and the volunteers performed the actions as defined by us in sequence in the EA environment setting in an empty hall. Generally, the movements of the human torso can be roughly divided into those in eight directions: Front, Back, Left, Right, Right-Front (RF), Right-Rear (RR), Left-Front (LF), and Left-Rear (LR). We found that leg movement could be covered in different directions. To ensure the consistency of movements in these directions, volunteers took steps in eight directions, and the results are shown in Figure 11a. The experimental results show that the recognition accuracy can be kept above 90% in the vertical path of visual distance. This is because Wi-Fi is less affected by multipath in the vertical transmission link, and the Doppler information is more sensitive, which makes the action information more abundant. CSI-F can have an average recognition accuracy of more than 85% in other directions. Although there are differences, the recognition effect of human movement is still significant. This thoroughly verifies that the CSI-F model can adapt to human actions from different directions.

2. Personal variety

Data collected from different people may differ due to their different behavior patterns. CSI-F combines Doppler energy to alleviate this problem. To evaluate the performance of CSI-F on a diversity of users, we trained the model on a data set of 10 people and then tested the data from a randomly selected volunteer. Figure 11b shows that the action-recognition accuracy of 10 people is kept near 90%, which thoroughly verifies the robustness of CSI-F for different people and proves the personnel-independence of CSI-F.

3. Feature difference

We compared three different features extracted from the original CSI measurements: amplitude, phase difference, and Doppler frequency shift. We inputted them into the deep learning model of the neural network. The recognition results, as shown in Figure 11c, show that the performance of CSI-F is superior to the amplitude and phase difference of CSI after denoising, with accuracy improvements of 8% and 5.2%, respectively. The improvement of CSI-F performance makes it unaffected by changes in transceiver layout, but this change will significantly impact the other two characteristics.

4. Diversity of equipment parameters

**Figure 12:**
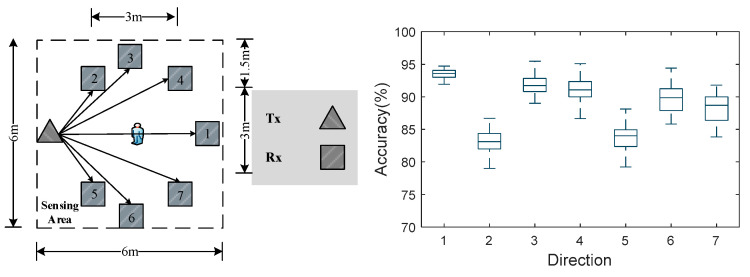
Identification accuracy at different deployment locations.

Generally, due to the different arrangements of items in other indoor environments, the deployment models of Wi-Fi will be diverse. Seven different device-deployment modes are set up in the experimental scene EB, and seven sensing areas are established. In other sensing scenes, we invited volunteers to perceive movement information by walking, and their deployment positions and perceptual results are shown in Figure 12.

Volunteers performed actions related to experimental settings at seven different device-deployment positions, and the experimental results are shown in Figure 12. When the two devices are in a straight line with the human, when they are deployed at location 1, the recognition accuracy reaches the highest, which is consistent with our experimental results in verifying the action’s direction. However, the recognition accuracy of locations 2 and 5 is the lowest, because the distance between the two devices is shorter and the distance from the people is greater, the perception area generated is small, the Fourier transform value is unstable, and the Doppler period-change information extracted is not sufficient. But overall, the recognition accuracy remains around 85% for different deployments, which is acceptable in most indoor scenarios.

5. Parameter difference

**Figure 13:**
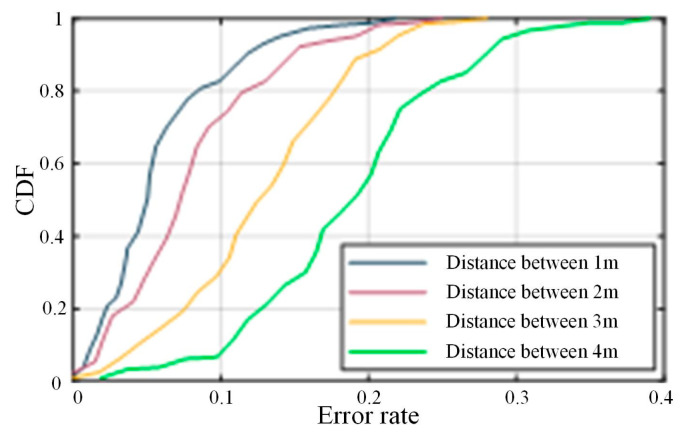
CDF of the error rate.

The distance between the transmitter and receiver also affects the recognition accuracy of the CSI-F system. To obtain the optimal detection distance, we separated the transmitter and receiver ends, using different distances, in the EB scene in the open hall, and tested the designed actions at each distance. Each action was executed in sequence. Figure 13 shows the cumulative distribution function (CDF) of the error rates under different scenarios. The *X*-axis represents the recognition error rate, and the *Y*-axis represents the percentage of the CDF. The highest accuracy was achieved when the devices were 1 m apart, with approximately 81% of the test data having an error rate of less than 10%. The recognition performance was worst when the spacing was 4 m; about 52% of the test data had an error rate of less than 20%. Generally, the CSI-F performance deteriorates as the distance between devices increases. However, 1 m spacing and 2 m spacing have similar recognition rates but provide a larger movement area, so it is appropriate to choose 2 m as the experimental deployment spacing in the verification experiment. However, 2M needs to meet the requirements of most users in indoor scenarios. Therefore, we increased the number of movement cycles based on spacing to explore a greater detection distance.

6. Difference in number of cycles

**Figure 14:**
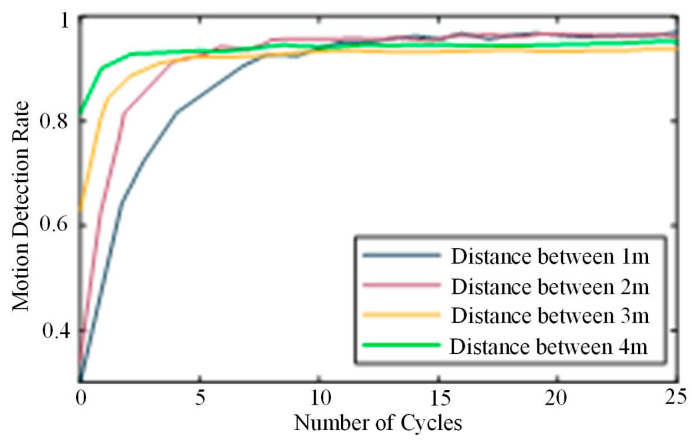
Differences in the number of cycles.

Different cycle times of the same action are set, respectively, to verify whether the cycle times can significantly impact CSI-F. In the EA scene in the open hall, the CSI data associated with the same action with different cycles, but the same equipment spacing, are collected. After multiple periodic CSI signals are superposed, the average value is taken to obtain a new continuous action signal. Then, the latest data is used as the input for human-action recognition. The experiment set the number of operation cycles as 0 to 25, and the equipment spacing was 2 m, 4 m, 6 m, and 8 m, respectively. Figure 14 shows the recognition accuracy of different cycle times at different equipment spacings.

It was found in the experiment that the accuracy rate of CSI-F can be maintained above 81% in the range of 4 m for one-off actions without a single periodicity. When the number of cycles of actions increases to five times, the distance between devices expands from 2 m to 8 m, and the influence of the recognition rate becomes less and less. The accuracy rate of recognition can converge to 90% within five cycles, which thoroughly verifies that CSI-F is perfectly adapted to cyclic actions, and the superposition of cycle information can improve the recognition accuracy of CSI-F. At the same time, CSI-F can provide a vast perception space in terms of deployment distance.

### 5.3. Comprehensive Performance Evaluation

#### 5.3.1. Evaluation of CSI-F’s Mobility

**Figure 15:**
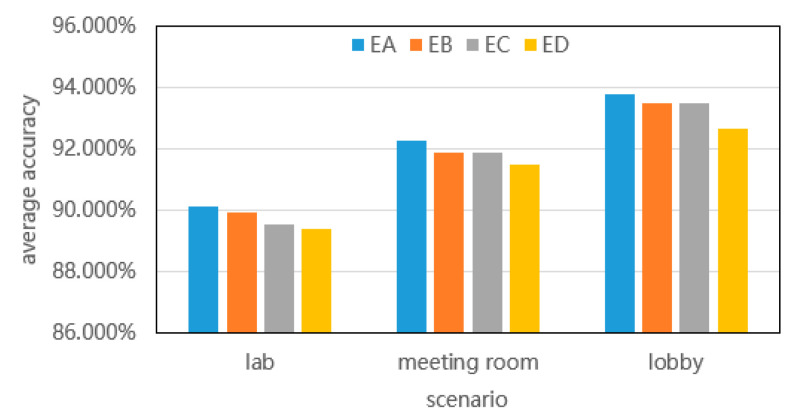
Identification results using different scenes.

Good training results were obtained by training the data collected in the four experimental scenarios of EA, EB, EC, and ED in the open hall. Similarly, data were collected in the laboratory and conference room to verify the model’s mobility. The experimental results are shown in Figure 15.

As can be seen from the experimental results in Figure 15, the CSI-F model has good recognition accuracy in EA, EB, EC, and ED in an open hall. This demonstrates that CSI-F shares the characteristics of primary and branch recognition tasks through multi-task mode, thus solving the problem of cross-scene recognition, and proves the auxiliary role of the CSI-F model branch task. At the same time, CSI-F trained in the open hall has a good effect on CSI data collected in EA, EB, EC, and ED in the laboratory and conference room, and the recognition results of the four scenarios in the same environment are not equal, which verifies the environmental mobility of the CSI-F proposed by us.

#### 5.3.2. Comparison of Different Classification Algorithms

**Figure 16:**
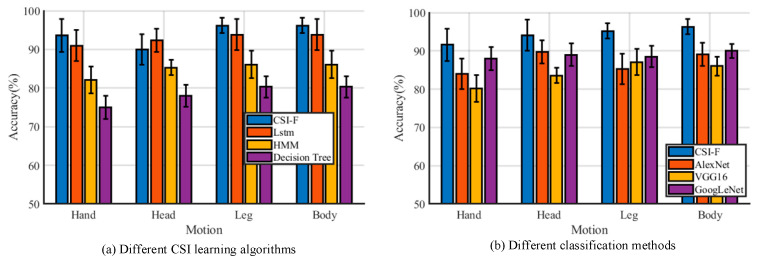
Different classifier algorithms.

To evaluate the performance of the classification method in CSI-F, 10,000 packages of movement data (including head, hand, and leg movements) obtained using 10 people were collected as training samples in an open hall environment, and the overall results designed were compared with LSTM, Hidden Markov Model (HMM), Decision Tree, and other classification algorithms described in previous work. The action-recognition effect of each classification method is shown in Figure 16a. It can be seen from Figure 16a that the motion recognition for head, hand, leg, and trunk movements, as well as other movements in different classification methods, can maintain high recognition accuracy. The accuracy levels of the CSI-F classification model, LSTM, HMM, and Decision Tree methods are 94.68%, 92.36%, 85.47%, and 78.51%, respectively. It shows that the improved neural-network deep-learning method can achieve better results than the traditional method after extracting features.

The most striking feature of CSI-F is that the model can learn from one task and transfer this knowledge to a separate task. Figure 16b lists three classical image recognition algorithms, and it can be seen that, compared with the other three algorithms (AlexNet, VGG16, and GoogLenet), the method in CSI-F has better adaptability and robustness with spectrum images.

#### 5.3.3. Comparison of Different Models

WiAct [28] used the correlation between torso movement and amplitude information in channel state information to classify different activities, and used an extreme learning machine to organize activity data. Wi-ATCN [29] combined causal and dilated convolution to ensure the integrity of CSI features, using self-attention mechanisms to obtain the most representative features of human behavior. ImgFi [30] converted CSI into images and used a convolutional neural network to recognize CSI images, which received a high recognition rate and reduced the complexity of the model. We have taken the average recognition accuracy as the index to evaluate the four methods, and the specific results are shown in Table 4.

We have recreated the above three methods as much as possible through online materials and compared them with CSI-F. The recognition effect is shown in Table 4. Among the four recognition methods, CSI-F has the highest recognition accuracy. The recognition rate of the other three methods, as to head and hand movements, is below 90%. Comparatively speaking, CSI-F demonstrates excellent performance. In summary, CSI-F can be applied for human action recognition in most indoor environments and can provide a more accurate recognition rate and outstanding robustness.

## 6. Conclusions

We have proposed a directional, independent and cross-scene environment-transfer mechanism for indoor personnel action-recognition in a Wi-Fi environment. This solves the problem of the significant difference in recognition accuracy caused by the need to correlate movement direction and recognition in different scenes in the same indoor environment. A discrete wavelet transform is introduced to filter out the signals irrelevant to human motion by eliminating phase offset from antenna diversity. Doppler frequency shift and motion energy transform values in signals are extracted by STFT and FFT, respectively, as the inputs of the CSI-F model, and the mutual assistance of motion-recognition tasks in different scenes is adopted. The CSI data of multiple tasks are input into the fusion network of CNN and GRU to achieve high-precision and cross-scene recognition through CSI feature-sharing and information fusion. The experimental results show that CSI-F can reach 94% recognition accuracy and is superior to many existing detection methods in accuracy and efficiency. Compared with the traditional sensing scheme, it can expand the coverage area of Wi-Fi sensing to 6 m. It can achieve similar recognition accuracy in different scenes of the same environment. This proves that CSI-F can realize cross-scene recognition and environment migration. Therefore, the method presented in this paper is feasible and efficient.

The follow-up work will be carried out in the following aspects: (i.) to further improve the generalization ability of the CSI-F model to improve the accuracy of action recognition in a multi-person environment; (ii.) to carry out feature-extraction of human movement speed, enrich the features of human movement, propose a more lightweight fast recognition scheme, and further improve the robustness of the algorithm.

## Figures and Tables

**Table 3:** Hyperparameters of the model.

Hyperparameter	Meaning	Hyperparameter	Meaning
$v_{j}^{l}$	Net activation	$B_{j}^{l}$	Deviation
⊗	Element-level multiplication	Loss_n_	Weighted sum of losses
$\beta_{n}$	Weight value of task n	tanh	Activation function
σ	Sigmoid function	ReLU	Activation function

**Table 4:** The differences between CSI-F and related work in action recognition based on Wi-Fi CSI.

Model	Evaluation	Head Motion	Hand Motion	Leg Motion	Body Motion
WiAct	Accuracy	0.750	0.865	0.90	0.931
Wi-ATCN	Accuracy	0.792	0.829	0.866	0.914
ImgFi	Accuracy	0.856	0.881	0.885	0.903
CSI-F	Accuracy	0.913	0.925	0.914	0.937

## Data Availability

Data are contained within the article.
